# Patient-derived orthotopic xenograft models recapitulate the peritoneal dissemination of pancreatic cancer and delineate its transcriptional and regulatory programs

**DOI:** 10.1186/s13046-026-03668-9

**Published:** 2026-02-11

**Authors:** Takaaki Furukawa, Kohei Kumegawa, Kenichi Miyata, Manabu Takamatsu, Asumi Iesato, Sumito Saeki, Liying Yang, Chikako Shibata, Tomoko Takahara, Kaoru Masuda, Takafumi Mie, Takeshi Okamoto, Tsuyoshi Takeda, Takashi Sasaki, Masato Ozaka, Miwa Tanaka, Shunji Takahashi, Tetsuo Noda, Ryoji Yao, Naoki Sasahira, Reo Maruyama

**Affiliations:** 1 Division of Cancer Epigenomics, Cancer Institute, Japanese Foundation for Cancer Research, 3-8-31, Ariake, Koto, Tokyo, Japan; 2 Department of Hepato-Biliary-Pancreatic Medicine, Cancer Institute Hospital of Japanese Foundation for Cancer Research, Tokyo, Japan; 3 Department of Genomic Medicine, Cancer Institute Hospital of Japanese Foundation for Cancer Research, Tokyo, Japan; 4 Cancer Bioscience/Oncotherapeutic Medicine, Tohoku University School of Medicine, Miyagi, Japan; 5 Cancer Cell Diversity Project, NEXT-Ganken Program, Japanese Foundation for Cancer Research, Tokyo, Japan; 6 Division of Pathology, Cancer Institute, Japanese Foundation for Cancer Research, Tokyo, Japan; 7 Department of Medical Oncology, Cancer Institute Hospital of Japanese Foundation for Cancer Research, Tokyo, Japan; 8 Director’s room, Cancer Institute, Japanese Foundation for Cancer Research, Tokyo, Japan; 9 Department of Cell Biology, Cancer Institute, Japanese Foundation for Cancer Research, Tokyo, Japan

**Keywords:** Pancreatic ductal adenocarcinoma, Peritoneal dissemination, Patient-derived organoids, Patient-derived orthotopic xenograft models, Single-cell multi-omics analyses

## Abstract

**Background & aims:**

The mechanisms of peritoneal dissemination in pancreatic ductal adenocarcinoma (PDAC) remain unclear partly owing to the lack of patient-derived models that recapitulate this process. This study aimed to establish an orthotopic model of PDAC peritoneal dissemination and to uncover the transcriptional and regulatory programs underlying this process.

**Methods:**

Organoids were established from primary pancreatic tumors and malignant effusions of patients with PDAC and orthotopically transplanted into the pancreas of immunodeficient mice to generate patient-derived orthotopic xenograft (PDOX) models. Subsequently, the organoids were rederived from pancreatic and peritoneal lesions of a representative model (PDOX12) and orthotopically reimplanted to assess the dissemination capacity. Single-nucleus RNA sequencing (snRNA-seq) and single-cell ATAC sequencing (scATAC-seq) were performed to analyze the tumors from these models.

**Results:**

The organoids that were derived from malignant effusions reproducibly generated peritoneal metastases after orthotopic implantation. To dissect this process more precisely, we focused on one representative model (PDOX12) and rederived organoids from its pancreatic and peritoneal lesions. These organoids generated matched PDOX models that differed only in dissemination potential when reimplanted orthotopically. The results of snRNA-seq revealed a distinct subpopulation enriched in the high-dissemination model, which was characterized by the coordinated activation of genes involved in cytoskeletal dynamics, extracellular matrix remodeling, and plasticity-related signaling—suggesting a dissemination-primed state. Integration with scATAC-seq identified STAT3, SMAD3, and SOX2 as potential upstream regulators of this gene program.

**Conclusions:**

This study established PDOX models that isolate the peritoneal dissemination phenotype and reveal the transcriptional and regulatory programs driving this process.

**Supplementary Information:**

The online version contains supplementary material available at 10.1186/s13046-026-03668-9.

## Introduction

Peritoneal dissemination is one of the most devastating manifestations of pancreatic ductal adenocarcinoma (PDAC), which frequently results in malignant ascites, intestinal obstruction, and rapid clinical decline [1]. After the liver, the peritoneum is the second most common metastatic site of PDAC. Peritoneal metastasis is present in approximately 9% of patients at the time of diagnosis and nearly 50% of patients at the time of death [2]. Despite its clinical importance, the biological mechanisms underlying peritoneal dissemination remain poorly understood, partly owing to the lack of patient-derived experimental models that faithfully recapitulate the stepwise process of peritoneal dissemination in vivo.

To understand the biology of peritoneal dissemination, the entire process in which tumor cells detach from the primary pancreatic lesion, survive in the peritoneal cavity, and colonize distant peritoneal sites needs to be modeled. However, existing models have inherent limitations. For example, genetically engineered mouse models (GEMMs), including Kras^G12D/+; Trp53^R172H/+; Pdx1-Cre (KPC) mice, can develop peritoneal dissemination [3, 4], highlighting the contribution of epithelial–mesenchymal transition (EMT), collective cell migration, and immunomodulation to this process [5–7]. However, the tumors are of murine origin and therefore fail to reflect the genetic diversity and clinical heterogeneity of human PDAC. Human-cell-line-based models have also been used [8–11]. Nevertheless, these models are typically generated by directly injecting cultured cells intraperitoneally, bypassing the critical steps of invasion and detachment from the pancreas. Patient-derived xenograft (PDX) models that were established from resected pancreatic tumors occasionally show peritoneal metastases [11, 12]. However, such events are incidental and have not been systematically studied as a dissemination model. Moreover, surgically resected PDAC specimens are usually obtained from patients without distant metastasis, further limiting their relevance to this disease state. Taken together, a human-derived model that reproduces the natural route of dissemination from the pancreas to the peritoneum has not been established.

To address this gap, the present study aimed to establish a patient-derived orthotopic model that reproduces the full metastatic cascade from the pancreas to the peritoneum. In principle, peritoneal lesions provide the most appropriate material for modeling dissemination because they represent tumor cells that have already acquired the ability to colonize the peritoneal cavity. However, they usually exist as small nodules or as free-floating malignant cells in ascites, making them technically unsuitable for direct transplantation as conventional PDXs. To overcome this limitation, patient-derived organoid (PDO) technology was used in this study, thereby enabling the expansion of even a small number of tumor cells from malignant ascites while preserving their genetic and phenotypic features [13, 14].

Building upon this platform, the transplantation of ascites-derived PDOs into the pancreas of immunodeficient mice was hypothesized to recapitulate the sequential processes of invasion, detachment, and peritoneal dissemination. Orthotopic implantation is critical for faithfully reproducing the natural progression of PDAC metastasis rather than intraperitoneal implantation. Here, patient-derived orthotopic xenograft (PDOX) models of PDAC that spontaneously develop peritoneal dissemination following orthotopic implantation were established. By integrating these models with single-nucleus RNA sequencing (snRNA-seq) and single-cell ATAC sequencing (scATAC-seq), candidate genes and regulatory programs that were potentially associated with peritoneal spread were identified. These findings highlight the utility of PDOX as a clinically relevant platform not only for recapitulating the natural history of PDAC dissemination but also for uncovering novel molecular targets that may guide the development of future therapeutic strategies.

## Materials and methods

### Collection of clinical specimens and establishment of organoids

Primary tumor tissues were obtained as residual specimens from endoscopic- ultrasound–guided fine-needle aspiration (EUS-FNA) performed for diagnostic purposes. Moreover, malignant effusion samples (ascites or pleural fluid) were collected from patients with advanced PDAC. Specimens were dissociated into single cells using the MACS Tumor Dissociation Kit and gentleMACS Dissociator (Miltenyi Biotec), in accordance with the manufacturer’s instructions. For ascites and pleural effusion samples, the drainage fluids were centrifuged and treated with the Red Blood Cell Lysis Solution (Miltenyi Biotec) to remove erythrocytes. The cell pellets were resuspended in basement membrane extract (BME; R&D Systems) at 4 °C. Thereafter, they were seeded to form domes in 6-well or 24-well plates (IWAKI), polymerized at 37 °C for 20–25 min, and overlaid with complete culture medium (500 µL per 50 µL BME dome). Organoids were cultured in complete medium initially and then in tumor medium. Those that proliferated for more than four weeks were considered successfully established. The medium was replaced every three to four days, and organoids were passaged depending on their growth. TrypLE (Thermo Fisher Scientific) and Y-27,632 (5 mM, Wako) were used during passaging. Organoids were also reestablished from orthotopic and peritoneal lesions of PDOX models using the same procedure.

### Organoid culture medium

The basic medium (AdDF+++) was prepared by supplementing Advanced DMEM/F12 (Gibco) with 5 mL penicillin–streptomycin (Gibco), 5 mL 10 mM HEPES (Gibco), and 5 mL GlutaMAX (Gibco) per 500 mL of medium. To generate complete medium (40 mL in total), 29.24 mL of AdDF+++ was mixed with 800 µL B27 supplement (50x, Gibco), 1,000 µL nicotinamide (400 mM, Sigma-Aldrich), 4 mL R-spondin 3 (100 µg/mL, PeproTech), 80 µL N-acetyl-L-cysteine (625 mM, Sigma-Aldrich), 40 µL Noggin (100 µg/mL, PeproTech), 400 µL EGF (5 µg/mL, PeproTech), 200 µL FGF10 (20 µg/mL, PeproTech), 40 µL Primocin (50 mg/mL, InvivoGen), 80 µL Y-27,632 (5 mM, Abmole), 40 µL A83-01 (500 µM, Tocris Bioscience), 4 mL Wnt3A (10% V/V), 40 µL Prostaglandin E2 (1 mM, Nacalai), and 40 µL Gastrin (10 µM, Tocris Bioscience). To generate tumor medium (40 mL), 29.68 mL of AdDF+++ with 800 µL B27 supplement (50x, Gibco), 1,000 µL nicotinamide (400 mM, Sigma-Aldrich), 4 mL R-spondin 3 (100 µg/mL, PeproTech), 80 µL N-acetyl-L-cysteine (625 mM, Sigma-Aldrich), 40 µL Noggin (100 µg/mL, PeproTech), 200 µL FGF10 (20 µg/mL, PeproTech), 40 µL Primocin (50 mg/mL, InvivoGen), 80 µL Y-27,632 (5 mM, Abmole), 40 µL A83-01 (500 µM, Tocris Bioscience), 4 mL Wnt3A (10% V/V), 40 µL, and 40 µL Gastrin (10 µM, Tocris Bioscience).

### Plasmid construction and lentivirus production

The pLenti-EGFP-ELuc plasmid was constructed by inserting the EGFP–Emerald Luciferase (ELuc) sequence into the pLenti CMV GFP Puro (658-5) vector (Addgene, #17448). The parental vector was digested with SalI and BamHI (Takara Bio), and the EGFP–ELuc insert was cloned using In-Fusion 5X HD Cloning Plus (Takara Bio) with 20–30 bp homologous sequences at both ends. The EGFP and ELuc coding sequences were fused via a 5′ linker sequence (5′- TCCGGCCGGA CTCAGATCTC GAGCTCAAGC TTCGAATTC-3′), and an SV40 poly(A) signal was added at the 3′ terminus. For lentivirus production, HEK293T cells were seeded at a density of 3 × 10⁶ cells per 10-cm dish. The following day, 2.5 µg of the lentiviral transfer plasmid, 1.25 µg of the pCMV-VSVG-RSV/Rev envelope plasmid, and 1.25 µg of the pMDLg/pRRE packaging plasmid were mixed in 250 µL Opti-MEM Reduced Serum Medium (Gibco). FuGENE HD Transfection Reagent (16 µL; Promega) was added to the mixture, which was gently vortexed, incubated for 15 min at room temperature, and then added dropwise to the HEK293T culture. The culture medium was replaced 16–24 h after transfection, and viral supernatants were collected at 48 and 72 h. The harvested medium was passed through a 0.45-µm filter (Millipore Millex-HV, SLHVR33RS), aliquoted, snap-frozen in liquid nitrogen, and stored at − 80 °C until use.

### Lentiviral transduction into organoids

Organoids were harvested from ongoing cultures and dissociated with TrypLE at 37 °C for 5–10 min through gentle pipetting in the presence of Y-27,632. Briefly, 1.0–2.0 × 10^5^ of single cells were resuspended in concentrated lentiviral particles encoding GFP–luciferase, supplemented with 10 µL polybrene (10 µg/mL). Spinoculation was performed at 3,000 rpm for 60–90 min at room temperature. Subsequently, the cells were incubated at 37 °C in 5% CO₂ for 9 h. Following transduction, the viral suspension was replaced with AdDF+++, and the cells were embedded in BME domes and overlaid with tumor medium. Puromycin selection was initiated 72–96 h after transduction and maintained for three to four days. Stable expression was confirmed by fluorescence microscopy for GFP.

### Mice

Six-week-old female NSG mice (The Jackson Laboratory Japan) were used. The mice were fed a radiation-sterilized diet (CLEA Japan) and housed under specific pathogen-free conditions in a room maintained on a 12-h light/dark cycle (lights on from 08:00 to 20:00; lights off from 20:00 to 08:00). All interventions were performed during the light cycle. All animal experiments were approved by the Institutional Animal Care and Use Committee of the JFCR (no. 22-01-2).

### Orthotopic transplantation

For orthotopic transplantation, six-week-old female NOD.Cg-PrkdcscidIl2rgtm1Wjl/SzJ (NSG) mice (The Jackson Laboratory Japan) were anesthetized by intraperitoneally administering a cocktail (200 µL per mouse) of medetomidine (0.75 mg/kg), midazolam (4 mg/kg), and butorphanol (5 mg/kg). A small incision was made in the left abdominal flank. Organoids (equivalent to approximately 1 × 10^6^ cells per mouse) expressing GFP-luciferase via lentiviral transduction were harvested and washed with AdDF+++. Afterward, they were dissociated into small fragments by gentle pipetting in TrypLE supplemented with Y-27,632 (5 mM, AbMole), and resuspended in 50 µL of BME gel (R&D Systems). The organoid suspension was then injected into the tail region of the pancreas using a 27-gauge insulin syringe. Successful injection was confirmed by the formation of a localized fluid bubble without intraperitoneal leakage. The abdominal wall was closed with sutures, and the skin was sealed with tissue adhesive. The mice were euthanized at designated time points for subsequent analyses. Tumor engraftment at the pancreatic and peritoneal sites was confirmed histologically or, when limited material was available, via the successful reestablishment of organoids from harvested tissues.

### Orthotopic and intraperitoneal retransplantation

Organoids were reestablished from the primary pancreatic lesions and peritoneal disseminated lesions of PDOX models. Dissociation, transplantation, and anesthesia were performed as described above. Mice were longitudinally monitored every 1–2 weeks using an in vivo imaging system (IVIS). For fluorescence imaging, the anesthetic mixture was first administered intraperitoneally, followed by intraperitoneal administration of luciferase. Luciferase was diluted 1:1 with PBS and administered at 200 µL per mouse. IVIS imaging was performed 10 min after luciferase administration. Mice that received orthotopic retransplantation were euthanized 70 days postimplantation, whereas those that received intraperitoneal retransplantation were euthanized 56 days postimplantation.

### Quantitative evaluation

During IVIS imaging, the primary pancreatic tumor and peritoneal disseminated lesions were visually identified, and luminescence was measured. At necropsy, the number of disseminated peritoneal nodules and the maximum diameter of each disseminated lesion were assessed macroscopically. The tumor weights of pancreatic and peritoneal lesions and the number of mice that developed peritoneal metastasis were recorded.

### Histologic evaluation

The tumor and organoid samples were fixed in 10% neutral buffered formalin, embedded in paraffin, sectioned in 4–5-µm sections, and stained with hematoxylin and eosin for histopathologic assessment. All slides were independently reviewed and evaluated by an experienced board-certified pathologist (Dr. Takamatsu).

### Histologic evaluation

Organoid and mouse tissue samples were fixed in 10% neutral buffered formalin (Wako) for 24 h. Thereafter, they were embedded in paraffin, sectioned at a thickness of 4–5 μm, and stained with Mayer’s hematoxylin and eosin.

### Cell viability assays

Organoids were dissociated into single cells and seeded at a density of 1,000 cells per well in 96-well plates (14 replicates for each organoid). The cells were cultured for four days in tumor medium. For viability assessment, 100 µL of CellTiter-Glo Luminescent Cell Viability Assay Reagent (Promega) was added to each well. The gels were disrupted using the Epoch2 Microplate Reader (BioTek). Then, the plates were incubated at room temperature for 30 min to allow complete cell lysis and ATP stabilization. Luminescence was measured using a multimode microplate reader (ARVO X3, PerkinElmer).

### Organoid migration assays

PDXO12A organoids cultured for 1 week were harvested by dissolving the gel using Cultrex Organoid Harvesting Solution (R&D Systems) and resuspended in 500 µL of tumor medium described above. At this stage, each of the following agents was added individually at two concentrations predetermined to avoid organoid cytotoxicity: Stattic (2 or 6µM; STAT3 signaling inhibitor; Selleck), SB431542 (0.5 or 1.5µM; TGF-β type I receptor/SMAD2/3 pathway inhibitor; Selleck), SIS3 (2 or 6µM; SMAD3 inhibitor; Cayman), Tiplaxin (0.5 or 1.5µM; SERPINE1 inhibitor; Selleck), TM5441 (3.3 or 10µM; SERPINE1 inhibitor; Selleck), and Anti-ANGPTL4 antibody (10 or 30µM; Selleck). Tumor medium without any adding compounds was used as a negative control. Next, 200 µL of BME was evenly distributed in each well of a 24-well tissue culture–treated plate (Corning) and allowed to polymerize at 37 °C for 20 min. The organoid suspension was then overlaid onto the solidified BME, with one well assigned per drug condition.

Time-lapse imaging was subsequently performed using the Incucyte Live-Cell Analysis System (Sartorius). Each well was divided into 36 fields of view, and images were acquired every 2 h for a total duration of 96 h. To quantify migratory activity, three representative fields of view per well were selected. GFP fluorescence-labeled organoids in each field were tracked using ImageJ, and migration distances during 0–24 h and 72–96 h were measured. The ratio of migration distance (r) during 72–96 h relative to that during 0–24 h was then calculated. Ratios obtained under each treatment condition were statistically compared with those of the negative control to evaluate inhibition of organoid migration.

### Single-cell and single-nucleus RNA sequencing

Single-cell RNA sequencing (scRNA-seq) and snRNA-seq were performed using the 10x Genomics Chromium platform. Thereafter, data processing was conducted using the Seurat (v5.1) pipeline. Low-quality cells were filtered out, and clustering was performed using principal component analysis (PCA) and uniform manifold approximation and projection (UMAP). Gene expression programs (GEPs) were identified by consensus nonnegative matrix factorization (cNMF), and recurrent programs (RPs) were defined on the basis of gene overlap across samples. The detailed methods are provided below.

### Single-cell ATAC sequencing

scATAC-seq was conducted using the ddSEQ Single-Cell Isolator (Bio-Rad), and data were analyzed using ArchR (v1.0.2) in accordance with a previous study [15]. Quality control was applied on the basis of transcription start site enrichment and fragment count. Peaks were called using MACS2, and motif accessibility was assessed using the cisBP database. The motif activity scores were correlated with the gene activity scores of cluster-specific gene sets that were derived from snRNA-seq to infer upstream transcriptional regulators. The detailed methods are provided below.

### Library preparation and sequencing for omics analyses

#### Bulk RNA-seq

Total RNA was extracted from PDOs using the RNeasy Mini Kit (Qiagen). RNA-seq libraries were generated using the SMARTer Stranded Total RNA Sample Prep Kit - HI Mammalian (Takara). Library quality and concentration were assessed with the TapeStation (Agilent), and pooled libraries were sequenced on the Illumina NextSeq 550.

#### Exome sequencing

Genomic DNA was extracted from PDOs using the DNeasy Blood & Tissue Kit (Qiagen). Exome libraries were prepared using the Illumina^®^ DNA Prep with Exome 2.0 Plus Enrichment (S) Tagmentation Set B (Illumina) according to the manufacturer’s protocol. Libraries were sequenced on an Illumina NextSeq 550 platform using 150-bp paired-end reads.

#### ScRNA-seq

Viable single-cell suspensions of PDOs were prepared and processed using the Chromium X instrument (10x Genomics) with the Chromium Next GEM Single Cell 3ʹ Kit v3.1, 3ʹ CellPlex Kit Set A, and 3ʹ Feature Barcode Kit (10x Genomics), following the manufacturer’s protocols. Four to five samples were multiplexed in each run using CellPlex sample barcodes. Library quality and concentration were evaluated by the TapeStation, and sequencing was performed on the NextSeq 550.

#### SnRNA-seq

Frozen PDOX tumor tissues were processed using the Chromium Nuclei Isolation Kit with RNase Inhibitor (10x Genomics). Isolated nuclei were then fixed and barcoded on the Chromium X using the Chromium Next GEM Single Cell Fixed RNA Sample Preparation Kit (10x Genomics) and the Chromium Fixed RNA Kit, Human Transcriptome (10x Genomics), in accordance with the manufacturer’s instructions. Four samples were multiplexed per run using fixed RNA barcodes. Library quality and concentration were evaluated by the TapeStation, and sequencing was performed on the NextSeq 550.

#### ScATAC-seq

scATAC-seq was performed as previously described [15] using the ddSEQ Single-Cell Isolator (Bio-Rad) together with the ddSEQ Single-Cell ATAC-Seq Library Prep Kit (Bio-Rad) according to the manufacturer’s protocol. Library quality and concentration were assessed with the TapeStation (Agilent), and pooled libraries were sequenced on the Illumina NextSeq 550.

### Data analysis

#### ScRNA-seq and snRNA-seq: preprocessing, quality filtering, clustering

We used the 10X Cell Ranger platform to generate snRNA-seq and scRNA-seq count matrix. The Seurat package (v5.1.0) was used for downstream analysis. Low-quality cells with ≥ 10% mitochondrial RNAs and < 500 or > 6000 features were filtered out. For filtered cells, the transcript count matrix was normalized to the total number of counts for the cell and multiplied by a scaling factor of 10,000. The normalized values were then natural-log transformed using Seurat’s NormalizeData function, followed by a linear transformation applied via the ScaleData function. Principal component analysis was performed using the RunPCA function, with the top 2000 highest variable features identified using the FindVariableFeatures function with the vst selection method. Subsequently, Seurat’s standard clustering procedures were performed using the FindNeighbors and FindClusters functions with the top 30 principal components (PCs) and a resolution of 0.2 for clustering of all PDOs and 0.6 for clustering per PDOs and in vivo model. Data visualization was achieved through the RunUMAP function, with the same PCs employed to identify clusters. The FindAllMarkers function with “only.pos = TRUE, logfc.threshold = 0.25” was employed to identify DEGs for each cluster. The genes with log2FC > 1 and adjusted P-value < 0.01 were identified as the cluster-specific genes.

#### ScRNA-seq: identification of recurrent transcriptional programs by cNMF

Consensus non-negative matrix factorization (cNMF) was performed per sample with a range of factorization ranks (4 ~ 9). Optimal k was determined for each dataset by inspecting stability metrics. For each factor, the top 200 genes by weight were retained, and Jaccard similarity between programs was computed. Hierarchical clustering was applied to identify groups of recurrent programs (RPs). Within each RP, gene groups common to one-third or more of the NMF programs were defined as genes of that RP. Overlap significance between the RPs and expression programs reported by Hwang et al. was calculated by R package GeneOverlap (v1.26.0).

#### ScATAC-seq: preprocessing, quality filtering, clustering

The hg19 genome assembly was used for the scATAC-seq analyses. The initial processing of scATAC-seq data to generate barcoded and aligned read bam files was performed using the ATAC-Seq Analysis Toolkit (Bio-Rad). Sequencing data were then processed by converting BAM files into fragment files using Rsamtools (https://bioconductor.org/packages/Rsamtools). R package ArchR (v1.0.2) was used for downstream analysis. Fragment files were loaded using the createArrowFiles function. Quality control analysis was conducted for each cell; cells with a transcription start site (TSS) enrichment score < 4 and number of unique fragments < 1500 were excluded. After creating arrow files for each sample, an ArchR project was generated containing all samples. To filter out doublets, addDoubletScores with k = 10, knnMethod = “UMAP,” LSIMethod = 1 was used. The addIterativeLSI function, with a genome-wide 500 bp tile matrix, was used to calculate the iterative LSI information. We clustered cells using addClusters (wrapper function of Seurat’s FindClusters). To run the uniform manifold approximation and projection (UMAP), we used the addUMAP function.

#### ScATAC-seq: peak calling and correlation analysis between gene score and motif score

For peak calling, we first generated pseudobulk replicates using the addGroupCoverages function, and then performed a MACS2 (v2.2.7.1) based peak call using the addReproduciblePeakSet function with the parameter method = “q,” cutOff = 0.05. The addPeakMatrix function was used to add the reproducible peak set to the ArchR project. Motif annotations from the cisBP databases were added to accessible chromatin regions using addMotifAnnotations function. Background peak sets were generated by the addBgdPeaks function, and chromVAR deviation matrices were calculated by the addDeviationsMatrix function. ArchR’s addModuleScore function was used to calculate module scores for scRNA-seq cluster-specific genes, and these scores were extracted from cell metadata and imputed based on weights using the imputeMatrix function. The motif deviation z-score matrices for cisBP were retrieved from the ArchR project using the getMatrixFromProject function and imputed using the same procedure. Pearson correlation coefficients were calculated for each module between module scores and all motif scores using the cor function.

### RNA-seq data analysis

To generate the expression count matrix, the raw reads were trimmed using Skewer (v. 0.2.2) to remove adapter sequences. The processed reads were mapped to the hg38 genome and counted using STAR (v2.7.8a). We used edgeR’s glmQLFTest (v3.32.1) to identify differential gene expression. As input, we used groups of control and intervention experiments with a simple design and a 0 intercept, “∼0 + Group.” First, we normalized the library sizes by calculating scaling factors with calcNormFactors(y, method = TMM). Next, we estimated the dispersion with estimateDisp(y, design = design, robust = TRUE) and fitted a generalized linear model with glmQLFit(y, design = design). Finally, we computed the log₂ fold changes and p-values using glmQLFTest.

### Publicly available data analysis

TCGA–PAAD RNA-seq data as a SummarizedExperiment object was downloaded using R package TCGAbiolinks’s GDCquery(), GDCdownload() and GDCprepare() functions. For survival analysis, we used R package survival’s survfit() function and package survminer’s ggsurvplot() function. The scRNA-seq data of primary and metastatic sites from the six patients with pancreatic cancer [16] was downloaded from NGDC (https://ngdc.cncb.ac.cn/; accession number PRJCA013942). All steps, including normalization, dimensionality reduction, and clustering, were performed as described above. Epithelial cells were annotated using the original cell-type annotations provided by the authors of the publication. Module scores representing the expression of the cluster 4 DEG signature were calculated for each epithelial cell using Seurat’s AddModuleScore function.

### Exome sequencing analysis

Paired-end sequencing reads were aligned to the human reference genome (GRCh38) using BWA-MEM with default parameters. PCR duplicates were identified and marked using Picard MarkDuplicates implemented in GATK, and duplicate metrics were collected for each sample. Base quality score recalibration (BQSR) was performed using GATK BaseRecalibrator with known single-nucleotide polymorphisms and indels obtained from public variant databases. Recalibrated BAM files were generated using GATK ApplyBQSR and indexed with SAMtools for downstream analyses. Somatic single-nucleotide variants and small insertions/deletions were identified from the recalibrated, duplicate-marked BAM files using GATK Mutect2 in tumor-only mode with the GRCh38 reference genome.Filtered somatic variants were annotated using ANNOVAR.

To assess mutation profiles of representative PDAC driver genes, annotated variant call format (VCF) files were further examined for KRAS, TP53, SMAD4, and CDKN2A. Variants were classified based on OncoKB annotations [17], and those annotated as oncogenic or likely oncogenic were retained. We focused on non-synonymous single-nucleotide variants, nonsense variants, and truncating insertions/deletions (frameshift indels). In addition, for TP53, in-frame insertions or deletions within the DNA-binding domain (amino acids 102–292) were also considered pathogenic alterations.

### Statistical analysis

Fisher’s exact test was used to evaluate statistical differences in the metastasis rate following orthotopic transplantation of PDOs into mice. Meanwhile, the Mann–Whitney U test was applied to assess statistical differences in the ModuleScore of RPs in scRNA-seq, cell viability of organoids, and tumor weight in mice. In in vitro migration assays, migration distance ratios among multiple treatment groups were assessed using the Kruskal–Wallis test followed by Holm-adjusted post hoc multiple comparisons. Statistical significance was considered at *P* < 0.05. All statistical analyses were performed using EZR software, version 1.40.

## Results

### Clinicopathological and transcriptomic characteristics of PDOs according to specimen origin

**Fig. 1:**
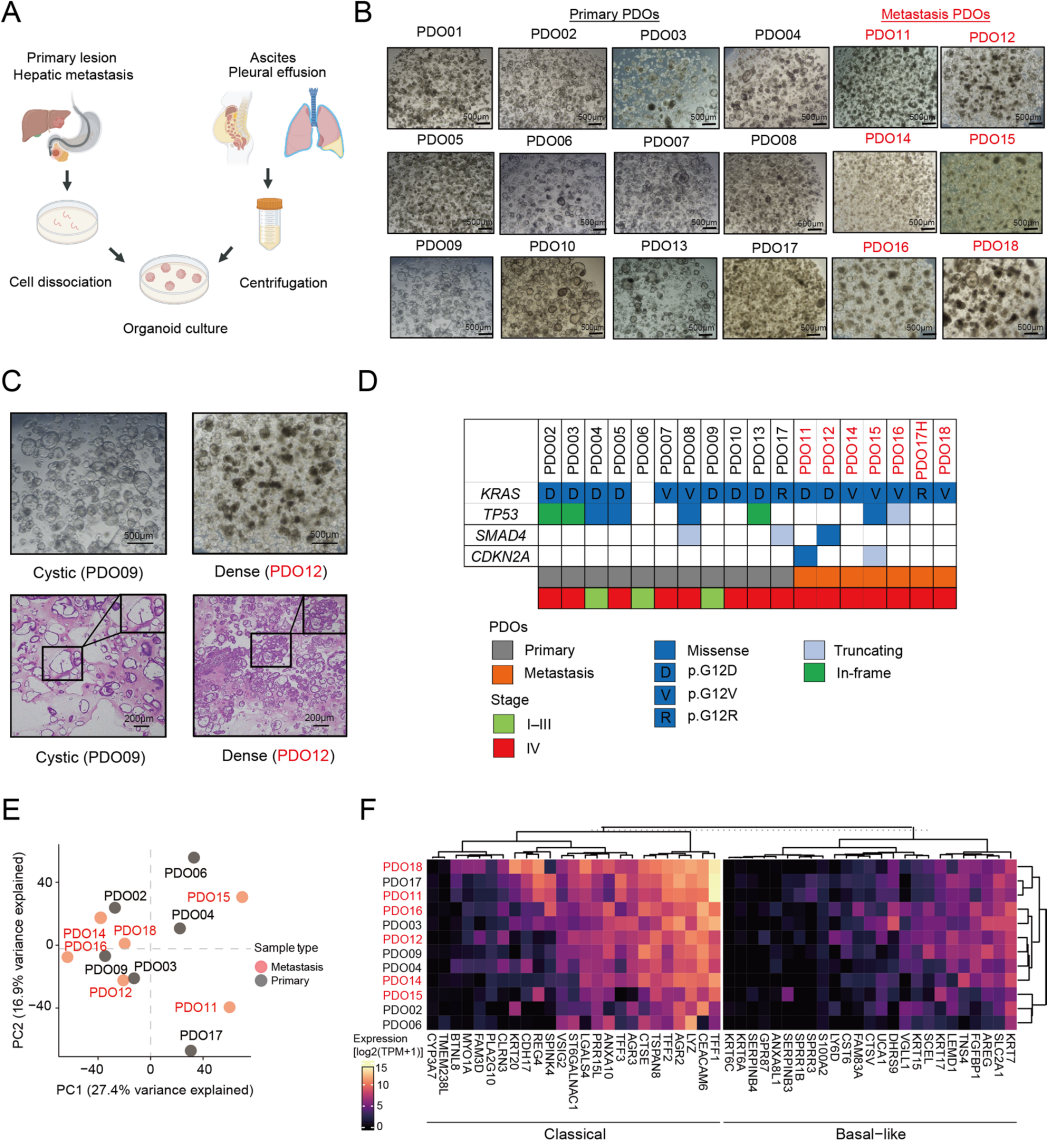
Morphological and transcriptomic characteristics of patient-derived organoids (PDOs). **A** Schematic for collecting clinical specimens and establishing organoids. **B** Representative images of the 18 PDOs. Scale bar: 500 μm. **C** Representative images of cystic (PDO09) and dense organoids (PDO12). Scale bar: 500 μm. **D** Mutation profiles in four core driver genes are shown across individual PDO lines. Only variants annotated as oncogenic or likely oncogenic according to OncoKB were included. KRAS mutation subtypes are indicated. Mutation types are color-coded. PDO origin and clinical stage at sampling are indicated at the bottom. **E** Principal component analysis (PCA) of 12 PDOs based on bulk RNA sequencing (RNA-seq) data. Each point represents one PDO, and the relative distances reflect transcriptomic similarities and differences among samples. **F** Heat map illustrating the differential expression patterns of classical- and basal-type signature genes across 12 PDOs based on bulk RNA-seq data. The rows represent each PDO, whereas the columns represent subtype-associated genes, with the color scale indicating relative expression levels

A panel of PDOs were generated from PDAC specimens that were obtained through various clinical sources to establish a foundation for modeling peritoneal dissemination (Fig. 1A; Table 1). This panel included 12 primary PDOs that were derived from pancreatic lesions obtained by EUS-FNA and seven metastasis PDOs that were derived from malignant effusions (five from ascites and one from pleural effusion) or a hepatic metastasis. The overall establishment success rates were 100% for metastasis PDOs and 57% for primary PDOs. Among the 18 cases, 15 cases were diagnosed with stage IV PDAC, two cases were diagnosed with stage III PDAC, and one case was diagnosed with stage I PDAC. Sixteen patients were chemotherapy-naïve at the sampling time.

**Table 1:** Clinicopathological characteristics of 18 patients with PDAC

Organoids	Age	Sex	Specimen	metastasis	cStage	Chemotherapy	Passage interval (day)	Morphology
PDO01	82	F	pancreatic lesion	lym	stage IV	No	7	dense
PDO02	57	F	pancreatic lesion	pul, oss	stage IV	No	7	cystic
PDO03	79	F	pancreatic lesion	peri	stage IV	No	7	cystic
PDO04	54	M	pancreatic lesion		stage III	No	14	cystic
PDO05	72	M	pancreatic lesion	hep	stage IV	No	6	dense
PDO06	53	F	pancreatic lesion		stage III	No	7	cystic
PDO07	69	M	pancreatic lesion	hep, pul, lym	stage IV	No	9	cystic
PDO08	86	M	pancreatic lesion	hep	stage IV	No	9	dense
PDO09	81	F	pancreatic lesion		stage I	No	14	cystic
PDO10	55	F	pancreatic lesion	hep	stage IV	No	9	cystic
PDO11	58	M	ascites	peri	stage IV	No	10	dense
PDO12	76	M	ascites	hep, lym, peri	stage IV	No	7	dense
PDO13	68	M	pancreatic lesion	hep	stage IV	No	7	cystic
PDO14	46	M	ascites	hep, lym, peri	stage IV	Yes	12	dense
PDO15	51	M	ascites	peri	stage IV	Yes	12	dense
PDO16	59	F	pleural effusion	hep, oss, ple	stage IV	No	10	dense
PDO17	54	M	pancreatic lesion	hep, lym, peri	stage IV	No	16	dense
PDO17H			hepatic metastasis				10	dense
PDO18	74	F	ascites	pul, peri	stage IV	No	12	dense

*PDAC* Pancreatic ductal adenocarcinoma, *lym* Lymph nodes, *hep* Liver, *oss* Bone, *peri* Peritoneum, *ple* Pleura, *pul* Lung

PDOs exhibited diverse morphologies under three-dimensional (3D) culture conditions (Fig. 1B and C). Primary PDOs (e.g., PDO09) frequently presented with cystic structures with thin walls and hollow lumens, whereas metastasis PDOs (e.g., PDO12) showed dense solid structures with thicker walls and focal necrosis (Table 1). The proliferation rate did not significantly differ between the two groups (*P* = 0.195), with passage intervals ranging from 6 days to 16 days for primary PDOs and seven days to 12 days for metastasis PDOs.

To further characterize the genetic background of the established PDOs, we examined somatic single-nucleotide variants in four representative PDAC driver genes—KRAS, TP53, SMAD4, and CDKN2A. KRAS mutations were detected in all PDOs except one. Oncogenic or likely pathogenic TP53 mutations were identified in eight PDOs, whereas SMAD4 and CDKN2A mutations were observed in three and two PDOs, respectively. Comparison between primary- and metastasis-derived PDOs did not reveal clear differences in the overall mutation profiles of these core driver genes. Notably, CDKN2A alterations were detected exclusively in two ascites-derived PDOs (Fig. 1D).

Next, the transcriptomic features of 12 representative PDOs were examined using bulk RNA sequencing. PCA did not reveal clear segregation between primary and metastasis PDOs (Fig. 1E). Similarly, classification according to the Moffitt basal-like/classical scheme [18] showed no consistent subtype bias (Fig. 1F), with all PDOs displaying higher expression of classical markers than basal-like markers. These findings suggest that although metastasis PDOs originate from tumor cells that have disseminated in vivo, such differences are not readily captured at the bulk transcriptome level under in vitro 3D culture conditions.

### PDOs recapitulate the transcriptional heterogeneity of primary pancreatic tumors

**Fig. 2:**
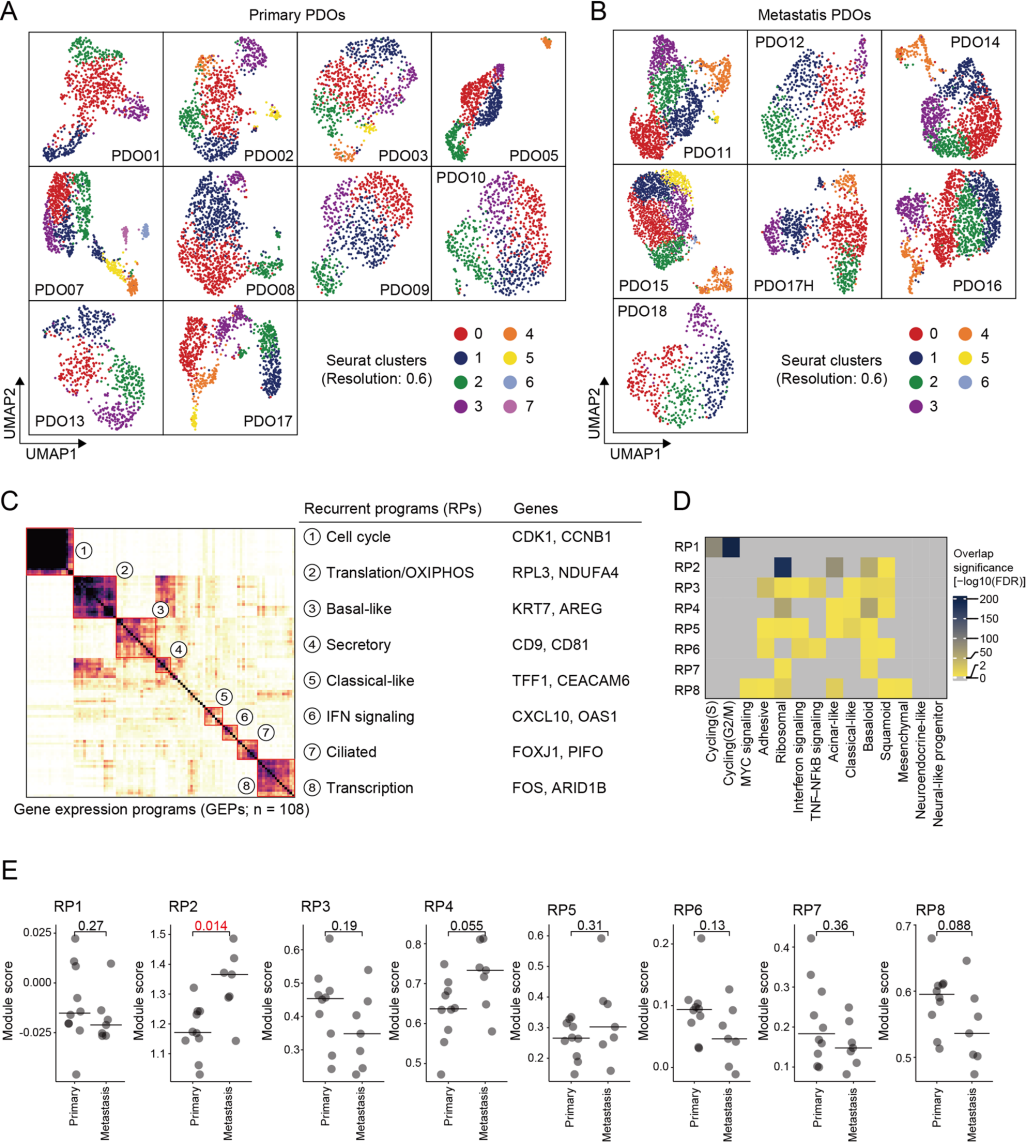
Patient-derived organoids (PDOs) recapitulate transcriptional heterogeneity of primary pancreatic ductal adenocarcinoma (PDAC) and reveal recurrent programs (RPs). **A** and **B** Uniform manifold approximation and projections (UMAPs) of single-cell transcriptomes from 17 PDOs, analyzed sample by sample. The cells were clustered within each PDO, and UMAPs are shown separately for primary (**A**) and metastasis PDOs (**B**). **C** Pairwise similarity analysis of gene expression programs (GEPs) identified by consensus nonnegative matrix factorization (cNMF) across the 17 PDOs. The heatmap shows the pairwise similarity matrix of 108 GEPs, which was quantified using the Jaccard index, with hierarchical clustering revealing eight major clusters defined as RPs shared across multiple PDOs. The eight RP assignments and representative genes for each RP are shown on the right. **D** Heatmap showing the degree of gene-level overlap between the eight RPs that were identified in this study and the 14 GEPs that were previously reported from single-cell transcriptomic analysis of PDAC patient specimens. **E** Differential analysis of RP activity between primary and metastasis PDOs

On the basis of their variable morphologies, PDOs likely comprise heterogeneous cell populations. As such, scRNA-seq was performed on 17 PDOs (10 primary PDOs and 7 metastasis PDOs) to characterize their transcriptional profiles with higher resolution. When all PDOs were jointly clustered, the cells segregated according to their PDO of origin (Supplementary Fig. 1A–F), which is consistent with previous reports [19] and confirms that PDOs preserve patient-specific transcriptional features. Clustering within each PDO revealed four to seven clusters per sample, with no apparent differences in the number of clusters between primary and metastasis PDOs. This finding indicates that both groups maintain comparable degrees of intra-tumoral heterogeneity (Fig. 2A and B).

To further delineate the transcriptional programs underlying this heterogeneity, cNMF was used to identify GEPs. For each PDO, the optimal rank was selected, and a total of 108 GEPs were identified across the 17 PDOs (Supplementary Data 1). Based on gene-level similarity, these GEPs were consolidated into eight RPs that were shared across multiple PDOs (Fig. 2C and Supplementary Data 2). These included programs enriched for the “classical” and “basal-like” signatures that were previously reported by Moffitt et al. [18]; moreover, they showed substantial overlap with GEPs that were identified in a previous scRNA-seq study of human PDAC tissues [20]. Specifically, 12 out of the 14 previously defined.

GEPs significantly overlapped with our RPs, whereas no RP overlapped with the neuroendocrine-like and neural-like progenitor programs (Fig. 2D). These findings confirmed that PDOs capture the spectrum of transcriptional states that are observed in clinical PDAC specimens.

Finally, the results of differential analysis of the eight RPs between groups revealed that RP2, which is associated with translation and oxidative phosphorylation (OXIPHOS), was significantly upregulated in metastasis PDOs (*P* = 0.014; Fig. 2E). This finding suggests that although PDOs as a whole faithfully recapitulate primary tumors’ transcriptional heterogeneity, metastasis PDOs may be characterized by enhanced biosynthetic and metabolic activity, potentially contributing to their peritoneal dissemination capacity in vivo.

### Establishment of a PDOX model of peritoneal dissemination using an ascites-derived PDO

**Fig. 3:**
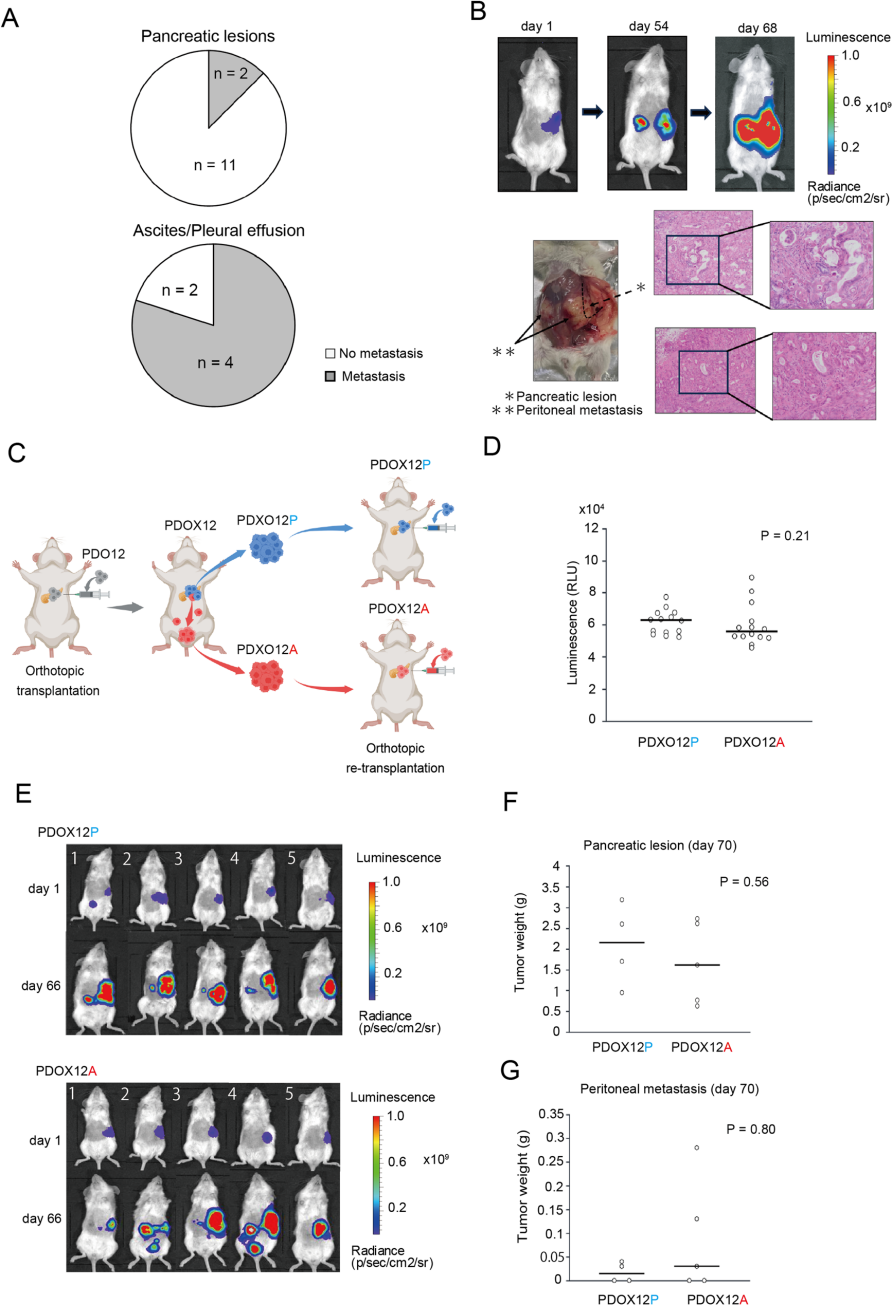
Differences in the peritoneal metastasis capacity of organoids derived from primary and peritoneal lesions in vivo. **A** Pie charts showing the proportions of mice with “metastasis” and “no metastasis” after orthotopic transplantation of patient-derived organoids (PDOs) established from primary pancreatic lesions (left) and ascites or pleural effusion (right). **B** In vivo imaging and histological analyses of a patient-derived orthotopic xenograft (PDOX) model after orthotopically transplanting PDO12 into the pancreas of NSG mice. Representative in vivo imaging system (IVIS) bioluminescence image showing pancreatic lesion and peritoneal metastases (top). Macroscopic appearance of the orthotopic pancreatic lesion and peritoneal metastases at necropsy (bottom left). Hematoxylin-and-eosin-stained images of the primary pancreatic lesion and peritoneal metastases (bottom right). **C** Orthotopic retransplantation scheme of organoids derived from the primary pancreatic lesion (PDXO12P) or peritoneal metastases (PDXO12A). **D** Dot plots of cell viability assays performed with CellTiter-Glo in 14 samples each of PDXO12P and PDXO12A. Each dot represents an individual sample, and horizontal bars indicate median values. Statistical significance was assessed using the Mann–Whitney U test. **E**–**G** In vivo imaging and tumor burden analyses of PDOX12P and PDOX12A. **E** Representative IVIS bioluminescence images. Dot plot comparing the weights of orthotopic primary pancreatic tumors (**F**) and peritoneal metastatic lesions (**G**) between PDOX12P and PDOX12A. Each dot represents an individual mouse, and horizontal bars indicate median values. Statistical significance was assessed using the Mann–Whitney U test

To determine whether the established PDOs possess the ability to induce peritoneal dissemination and to establish an in vivo model that recapitulates this process, we orthotopically transplanted seven PDO lines into the pancreas of NSG mice (four mice per line), including four primary PDOs and three metastasis PDOs (Supplementary Fig. 2A). During the observation period, seven mice died, most of which had received metastasis PDOs (Supplementary Fig. 2B). Tumor engraftment was confirmed in 19 mice at necropsy. These engrafted tumors were referred to as PDOX models. Peritoneal dissemination was more frequently observed in mice transplanted with metastasis PDOs than in those transplanted with primary PDOs. Overall, six out of the 19 PDOXs developed metastases, including four out of six engrafted mice transplanted with metastasis PDOs (66.7%) compared with two out of 13 mice transplanted with primary PDOs (15.4%; *P* = 0.046) (Fig. 3A, Supplementary Fig. 2B, and Supplementary Table 1).

Among these six PDOXs, five exhibited peritoneal metastases and two showed lung metastases. The peritoneal lesions could be detected by fluorescence imaging in four cases (Fig. 3B). Moreover, organoids were successfully reestablished from three of them (data not shown), demonstrating the feasibility of serial modeling. By contrast, lung metastases were not detected by in vivo imaging but were histologically identified at necropsy (Supplementary Fig. 2C). The results of histological analysis revealed that orthotopic pancreatic tumors and peritoneal metastatic lesions retained ductal morphology with prominent stromal components, closely recapitulating the histopathological architecture of human PDAC.

We focused on PDO12 and performed serial transplantation to generate a PDOX model with enhanced capacity for peritoneal dissemination. Organoids were reestablished from the orthotopic pancreatic tumor (PDXO12P) and from a peritoneal metastatic lesion (PDXO12A) of the same mouse (PDOX12). Then, they were orthotopically retransplanted into the pancreas of NSG mice (five mice per group) to compare their growth in the pancreas and their ability to generate peritoneal dissemination (Fig. 3C). In vitro, CellTiter-Glo assays revealed no significant difference in the proliferative capacity between PDXO12P and PDXO12A (Fig. 3D). Consistently, both groups efficiently engrafted in the pancreas, and no significant difference was observed in luminescence or primary tumor weight at necropsy (PDXO12P, 2.3–3.6 × 10^9 p/sec/cm^2^/sr and 0.95–3.19 g; PDXO12A, 0.3–3.7 × 10^9 p/sec/cm^2^/sr and 0.63–2.74 g; *P* = 0.55 and 0.56, respectively) (Fig. 3E and F, and Supplementary Fig. 3A and B). Nevertheless, peritoneal dissemination was more prominent in the PDXO12A group than in the PDXO12P group: although the dissemination frequency was comparable, mice transplanted with PDXO12A developed markedly larger metastatic nodules, with some exceeding 1.5 × 10^9 in luminescence p/sec/cm^2^/sr and 0.2 g in size, compared with those transplanted with PDXO12P (Fig. 3E and G, Supplementary Fig. 3A and B, and Supplementary Table 2).

We also performed direct intraperitoneal transplantation of PDXO12P and PDXO12A to assess whether this difference reflected an enhanced colonization capacity (five mice per group; Supplementary Fig. 3C). In this setting, all mice developed peritoneal tumors. However, no significant differences in luminescence or tumor weight were observed (PDXO12P, 1.7–6.5 × 10^9 p/sec/cm^2^/sr and 0.39–1.57 g; PDXO12A, 2.0–5.7 × 10^9 p/sec/cm^2^/sr, 0.48–1.30 g; *P* > 0.99 and = 0.69, respectively) (Supplementary Fig. 3D and E). Collectively, these results indicate that organoids that were rederived from peritoneal metastases (PDXO12A) do not exhibit increased proliferative capacity in vitro or within the pancreas, nor do they show superior engraftment ability when transplanted directly into the peritoneum. However, when reintroduced into the pancreas, PDXO12A displayed a greater capacity to generate large secondary lesions in the peritoneum compared with their primary-tumor-derived counterparts (PDXO12P). Taken together, PDXO12A represents an organoid line with an enhanced ability to recapitulate the sequential process of dissemination from the pancreas to the peritoneum. Thus, the PDOX12 lineage, which encompasses dissemination-competent and less-competent derivatives, provides a powerful platform for identifying subtle molecular determinants of peritoneal spread.

### In vivo modeling of PDOs identifies a cell population driving peritoneal metastasis

**Fig. 4:**
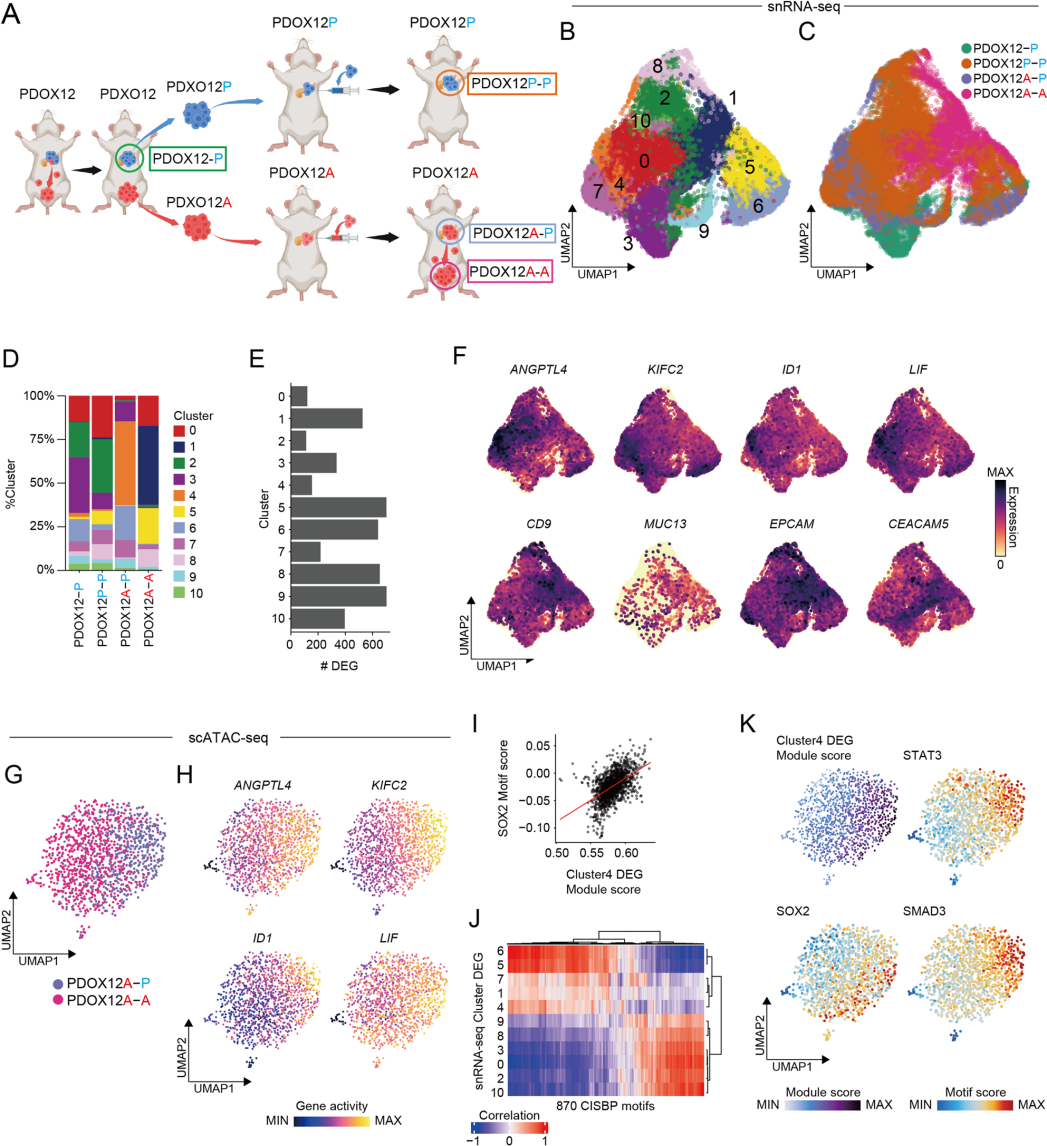
Single-cell multiomic analysis of the PDOX12 lineage revealing dissemination-associated transcriptional programs. **A **Schematic overview of the PDOX12 lineage used for single-nucleus RNA sequencing. The colors indicate the tissue origin: green circle, primary pancreatic lesion from PDOX12 (PDOX12-P); yellow circle, primary pancreatic lesion from PDOX12P (PDOX12P-P); gray circle, primary pancreatic lesion from PDOX12A (PDOX12A-P); violet circle, peritoneal metastatic lesion from PDOX12A (PDOX12A-A). **B** and **C** Uniform manifold approximation and projection (UMAP) visualization of 21,523 single-nucleus transcriptomes from the four tumors. Cells colored by cluster identity (**B**) and sample of origin (**C**). **D** Proportion of each cluster across the four tumors. **E** Number of cluster-specific differentially expressed genes (DEGs). **F** Representative examples of cluster-4 and cluster-1-specific DEGs expression on UMAPs. **G** UMAP visualization of single-cell ATAC sequencing (scATAC-seq) of PDOX12A-P and PDOX12A-A. Cells colored by sample of origin. **H** UMAPs colored by gene-activity scores for cluster-4-specific DEGs. **I**–**K** Integrative analysis of chromatin accessibility and transcriptional activity using scATAC-seq data. Correlations were calculated between the module scores (derived from gene activity scores of cluster-specific DEGs) and the motif activity scores across all single nuclei. **I** Example scatter plot showing the correlation between cluster-4-specific DEG module score and SOX2 motif activity score at the single-cell level. **J** Heatmap showing the correlation coefficients between each DEG module score and all transcription factor motif activity scores, identifying regulatory modules with shared motif-association patterns. **K** UMAP illustrating the representative motif activities correlated with the cluster-4-specific DEG modules

Building on the PDOX12 lineage, we next sought to identify transcriptional states that were associated with metastatic competence. To this end, snRNA-seq was performed on four tumors that were derived from this lineage: PDOX12-P (the original orthotopic tumor), PDOX12P-P and PDOX12A-P (orthotopic tumors from mice that were reimplanted with PDXO12P or PDXO12A, respectively), and PDOX12A-A (the peritoneal metastasis from the PDOX12A mouse) (Fig. 4A). The primary comparison between PDOX12A-P and PDOX12P-P (orthotopic tumors from mice with relatively high versus low peritoneal dissemination propensity) was designed to capture transcriptional programs associated with dissemination initiation. In parallel, comparing PDOX12A-P with PDOX12A-A provided insights into the consequences of dissemination, revealing how tumor cells adapt after colonizing the peritoneal cavity.

After quality filtering, high-quality single-nucleus transcriptomes were obtained from 21,523 cells (Supplementary Fig. 4A–C). Clustering analysis identified 11 distinct clusters across the four tumors (Fig. 4B and **C**). The overall cluster distributions revealed that PDOX12-P and PDOX12P-P exhibited highly similar patterns, consistent with their shared derivation from pancreatic lesions without prior peritoneal passage. By contrast, PDOX12A-P and PDOX12A-A displayed divergent distributions, which reflects the transcriptional consequences of prior dissemination and peritoneal colonization (Fig. 4D). Notably, cluster 4 was specifically enriched in PDOX12A-P, which represents a cell population that is potentially involved in dissemination initiation from the pancreas, whereas cluster 1 was predominantly enriched in PDOX12A-A, which suggests a population that is adapted for colonization and survival within the peritoneal cavity.

To investigate the basis of these shifts, we subsequently identified cluster-specific differentially expressed genes (DEGs; log2FC > 1, adjusted *P* < 0.01; Fig. 4E). Cluster 4, which was enriched in PDOX12A-P, showed coordinated upregulation of genes involved in extracellular matrix remodeling and invasion (e.g., *SERPINE1* and *ANGPTL*4), cytoskeletal dynamics (e.g., *KIF* and *MYO* family members), and developmental or plasticity-related signaling (e.g., *WNT11*, *JUN*, *ID1*, and *LIF*; Fig. 4F and Supplementary Data 3). These features suggest that cluster 4 represents a population that is transcriptionally primed for the initiation of peritoneal dissemination. By contrast, cluster 1, which was dominant in PDOX12A-A, contained numerous genes encoding membrane-associated, secretory, and lysosomal proteins, together with epithelial markers (e.g., EPCAM and CDH1), and enzymes related to lipid metabolism and cholesterol biosynthesis (Fig. 4F and Supplementary Data 3). Collectively, these features indicate differentiated epithelial cells with a secretory and membrane-forming program, which is consistent with adaptation to the peritoneal microenvironment.

To further explore the potential clinical relevance of the cluster-4 gene program, we analyzed publicly available transcriptomic datasets. Using bulk RNA-seq data from The Cancer Genome Atlas (TCGA) pancreatic ductal adenocarcinoma (PAAD) cohort, patients were stratified into two groups based on the average expression level of cluster-4 genes. Kaplan–Meier analysis revealed that patients with higher expression of cluster-4 genes showed a significantly worse overall survival compared with those with lower expression (Supplementary Fig. 4D). We further examined single-cell RNA-seq datasets comprising paired primary tumors and liver metastases from six PDAC patients [16]. In each dataset, a subset of tumor cells exhibiting high expression of the cluster-4 gene signature was detected. These observations suggest that this dissemination-associated transcriptional program can be observed across patients and disease sites (Supplementary Fig. 4E–G). Together, these analyses support the clinical relevance of the cluster-4 gene program identified in the PDOX model.

To elucidate the upstream regulatory mechanisms governing these gene programs, scATAC-seq was performed on PDOX12A-P and PDX12OA-A (Fig. 4G and Supplementary Fig. 5A and B). The gene activity scores, which infer transcriptional output from chromatin accessibility profiles, were calculated for each cluster-specific DEG set. The scores for cluster-4-specific genes were higher in cells derived from PDOX12A-P (Fig. 4H). We calculated the correlations between the motif accessibility scores and the module scores derived from cluster-specific DEG sets across individual cells to further link the regulatory activity with the transcriptional output. This analysis stratified the snRNA-seq DEG clusters into four groups with similar motif correlation patterns (Fig. 4I and J and Supplementary Data 4), suggesting that each group may be under a shared transcriptional control. Among them, the DEG module of cluster 4 showed strong and specific correlations with motifs for several transcription factors, including SOX2, STAT3, and SMAD3 (Fig. 4I and K and Supplementary Fig. 4F and G), all of which have been implicated in epithelial plasticity and metastatic progression [21–23]. These findings suggest that these transcription factors may constitute upstream regulators of the dissemination-primed gene program enriched in cluster 4.

To functionally assess the contribution of cluster-4–associated pathways, we performed in vitro migration assays using PDXO12A organoids treated with pharmacological inhibitors targeting candidate upstream regulators and effector pathways. Inhibition of TGFβ signaling with SB431542 significantly reduced organoid migratory activity, whereas blockade of STAT3 signaling showed a trend toward suppression without reaching statistical significance after multiple-comparison correction (Supplementary Fig. 5H–J and Supplementary Data 5). Together, these results provide supportive functional evidence for a potential contribution of TGFβ-signaling, and suggest a possible involvement of STAT3-related pathways, in the dissemination-associated phenotype identified by single-cell analyses.

## Discussion

The biology of peritoneal dissemination, one of the most devastating manifestations of PDAC, remains poorly understood in part because of the lack of patient-derived models that authentically reproduce this process. In the present study, PDOX models were established by transplanting organoids derived from malignant ascites into the pancreas of immunodeficient mice. These models faithfully recapitulated the histological architecture of human PDAC and spontaneously developed peritoneal dissemination, thereby filling a critical gap between GEMMs and traditional xenografts using established cell lines. Our PDOX model provides a clinically relevant and experimentally tractable platform for studying PDAC dissemination mechanisms by preserving patient-specific features and enabling controlled genetic manipulation.

Extensive studies using GEMMs, including the KPC model, have revealed key mechanisms underlying PDAC dissemination, including EMT [24], polyclonal metastatic seeding [5], immunosuppressive microenvironmental remodeling [7], and stromal interactions [6] that facilitate peritoneal spread. Although these models have been invaluable for dissecting tumor–stroma and immune crosstalk within an intact murine microenvironment, their murine origin limits their ability to capture the genetic diversity and clinical heterogeneity of human PDAC. By contrast, several mouse models using human cell lines or patient-derived specimens have been developed. However, most rely on subcutaneous or intraperitoneal tumor cell injection, bypassing the critical steps of invasion and detachment from the pancreas [25]. A previous study that was conceptually similar to ours established PDXs from malignant ascites. However, these PDXs were subcutaneously implanted and did not develop peritoneal dissemination [26], suggesting that orthotopic implantation may be required to reproduce this process. Conversely, orthotopic PDX models generated from resected primary PDAC tissues have occasionally exhibited peritoneal metastases [11, 12]. Nevertheless, such findings were incidental and have not been systematically analyzed as a dissemination model. Together, these limitations underscore the absence of a patient-derived orthotopic model that faithfully recapitulates the sequential process of dissemination from the pancreas to the peritoneum.

Through snRNA-seq analysis of this system, distinct transcriptional programs associated with different steps of metastasis were identified in the present study. In particular, pancreatic tumors derived from peritoneal-origin organoids (PDXO12A) were enriched for a cell population characterized by coordinated upregulation of genes involved in cytoskeletal dynamics, stress response, extracellular matrix remodeling, and immune modulation—molecular processes that collectively promote invasion and early dissemination [27, 28]. Among them, the well-known metastasis-associated genes, including *SERPINE1*, *ANGPTL4*, and *LIF*, were prominently expressed. SERPINE1 enhances cell survival, migration, and invasion [29]. Meanwhile, ANGPTL4 drives motility and EMT [30–32] while regulating vascular permeability and angiogenesis [32, 33]. Acting through the JAK/STAT3 pathway, LIF promotes tumor migration and establishes an immunosuppressive and prometastatic microenvironment [34]. Analysis of public transcriptomic datasets further suggested the clinical relevance of this program: a high cluster-4 gene expression was associated with poorer overall survival in the TCGA PDAC cohort, and cells exhibiting this signature were detected across paired primary and metastatic lesions in independent single-cell datasets. Together, these findings suggest that this transcriptional module represents a dissemination-primed state within the primary tumor, providing a valuable resource for future mechanistic studies of metastatic initiation.

The integrative analysis with scATAC-seq further highlighted STAT3, SMAD3, and SOX2 as candidate upstream regulators of the dissemination-associated gene program, which is consistent with their known roles in stemness and lineage plasticity. STAT3 signaling has previously been implicated in promoting metastatic progression, including peritoneal dissemination, in KPC mouse models of pancreatic cancer [7] and in patient-derived orthotopic models of ovarian cancer [35], supporting its potential relevance in this context. STAT3 acts in conjunction with the proinflammatory cytokine Interleukin-6 to promote *ANGPTL4* expression in cancer-associated fibrosis [36]. SMAD3 forms a complex with SMAD4 and binds to the CAGA boxes in the *SERPINE1* promoter, which regulates its transcription [37]. LIF expression is driven by a cooperative complex of STAT3, SMAD3, and SOX2, thereby sustaining cancer stem cell traits and tumor growth [38]. Functional assays further provided experimental support for this regulatory framework: pharmacological inhibition of TGFβ signaling significantly reduced the migratory capacity of dissemination-competent organoids in vitro, while inhibition of STAT3 signaling showed a consistent, albeit more modest, suppressive trend. While these findings are based on short-term pharmacological perturbation in an in vitro setting, the experiments were exploratory in nature and lend functional support to the regulatory network inferred from single-cell analyses.

A particularly striking aspect of our findings is the cellular plasticity revealed by the PDOX system. In vitro, PDXO12P and PDXO12A organoids exhibited nearly indistinguishable transcriptional and proliferative profiles. However, their latent differences were unmasked when placed in vivo: PDXO12A demonstrated stronger peritoneal dissemination, accompanied by clear transcriptional divergence. Upon colonizing the peritoneum, the tumor cells underwent another major state transition, adopting distinct expression programs adapted to the new environment. Remarkably, these cells reverted to a similar transcriptomic state when re-cultured as 3D organoids, again concealing the differences observed in vivo (data not shown). These observations suggest that tumor cell state transitions are driven by contextual cues from the tumor microenvironment. In the PDOX model, human cancer cells interact with murine fibroblasts, endothelial cells, and extracellular matrix components, and both physical constraints and direct cell–cell or cell–matrix interactions may initiate epigenomic and phenotypic reprogramming. In the present study, single-nucleus RNA sequencing was performed using human-specific probes, and the analyses were therefore restricted to tumor-intrinsic transcriptional programs. Although murine stromal nuclei were captured, their transcriptomes could not be reliably profiled and were excluded during data processing. Consequently, stromal-derived signals that may act as upstream triggers of cancer cell plasticity could not be directly assessed. Future studies employing mouse-specific probes or cross-species single-cell sequencing approaches [39] may enable systematic analysis of tumor–stroma interactions within this framework. Another limitation of the PDOX model is the use of immunodeficient mice, which precludes evaluation of immune-mediated contributions to peritoneal dissemination. Given accumulating evidence for immune involvement in metastatic progression [7], this represents an important distinction from immunocompetent models such as KPC mice. Incorporation of humanized immune systems into the PDOX platform may help overcome this limitation and further extend the utility of the model.

## Conclusions

We developed a PDOX model that faithfully reproduces the clinical and histological features of PDAC peritoneal dissemination. This model offers both conceptual and practical advances. Conceptually, it highlights cancer cells’ dynamic plasticity and their context-dependent behavior. Practically, it serves as a versatile experimental platform for the functional interrogation of metastasis-associated programs. Future studies leveraging this system may clarify the epigenomic mechanisms that confer metastatic potential and ultimately guide the development of new therapeutic strategies targeting peritoneal dissemination in PDAC.

## Supplementary Information

Supplementary Material 1.

Supplementary Material 2.

Supplementary Material 3.

Supplementary Material 4.

Supplementary Material 5.

Supplementary Material 6.

## Data Availability

The bulk RNA-seq, scRNA-seq, snRNA-seq, and scATAC-seq datasets generated in this study have been deposited in the Gene Expression Omnibus (GEO) under accession numbers GSE318411, GSE318412, and GSE318413. The R code used to reproduce the results is publicly available at https://github.com/KoheiKumegawa/PDOX_PDAC.

## Abbreviations

bulk RNA-seq: Bulk RNA sequencing
cNMF: Consensus non-negative matrix factorization
DEGs: Differentially expressed genes
EMT: Epithelial–mesenchymal transition
EUS-FNA: Endoscopic ultrasound-guided fine-needle aspiration
GEMMs: Genetically engineered mouse models
IVIS: In vivo imaging system
KPC: Kras^G12D/+; Trp53^R172H/+; Pdx1-Cre
NSG: NOD.Cg-Prkdc^scid Il2rg^tm1Wjl/SzJ
OXPHOS: Oxidative phosphorylation
NC: Negative control
PCA: Principal component analysis
PDAC: Pancreatic ductal adenocarcinoma
PDO: Patient-derived organoid
PDOX: Patient-derived orthotopic xenograft
PDXO12A: Organoid derived from the peritoneal metastatic lesion of PDOX12
PDXO12P: Organoid derived from the orthotopic pancreatic lesion of PDOX12
PDOX12-P: The primary pancreatic lesion of the original PDOX12
PDOX12A-A: The peritoneal metastatic lesion derived from PDXO12A
PDOX12A-P: The primary pancreatic lesion derived from PDXO12A
PDOX12P-P: The primary pancreatic lesion derived from PDXO12P
RP: Recurrent program
scATAC-seq: Single-cell ATAC sequencing
scRNA-seq: Single-cell RNA sequencing
snRNA-seq: Single-nucleus RNA sequencing
