# Supplementary material for: Patient-derived orthotopic xenograft models recapitulate the peritoneal dissemination of pancreatic cancer and delineate its transcriptional and regulatory programs
Source: J Exp Clin Cancer Res. 2026 Feb 11;45:69. doi: 10.1186/s13046-026-03668-9 (PMC12998334; doi:10.1186/s13046-026-03668-9)
Supplement: Supplementary file 6 — Supplementary Material 6. [file 13046_2026_3668_MOESM6_ESM.pdf]

Supplementary Figure 1

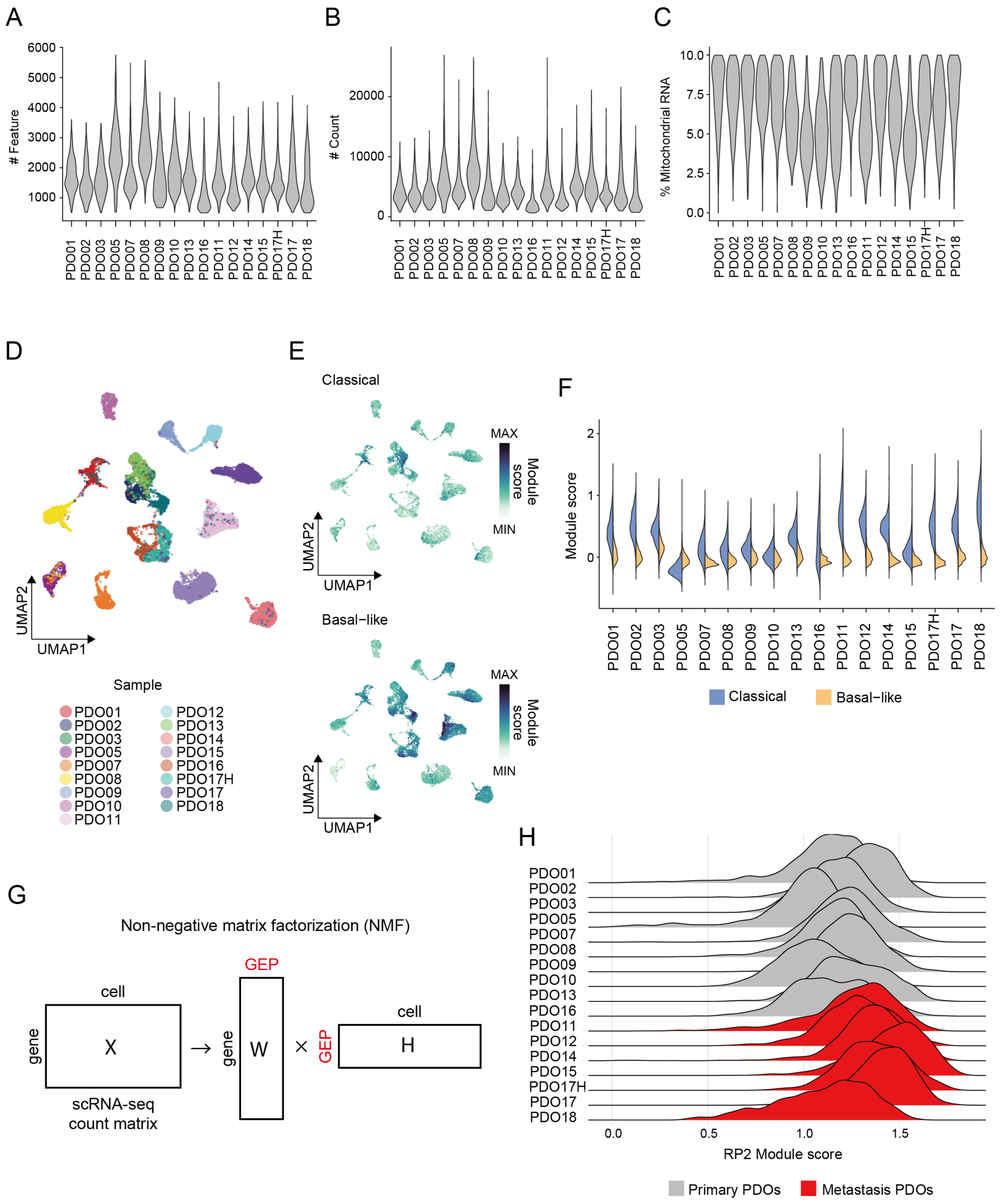

Supplementary Figure 1. Single-cell RNA (scRNA-seq) sequencing data of 17 PDOs, related to Fig. 2. (A–C) Quality control metrics for all analyzed PDO samples. Distribution of detected features per cell (A), distribution of total UMI counts per cell (B), and percentage of mitochondrial RNA counts per cell (C). Each violin plot represents the distribution of individual cells within each PDO sample. (D) Uniform Manifold Approximation and Projection (UMAP) visualization of all single cells combined across PDO samples. Each color indicates a distinct

PDO. (E) UMAP plots showing module scores for classical and basal-like gene signatures. (F) Violin plots showing the distribution of classical and basal-like module scores across individual cells in each PDO. (G) Schematic overview of the non-negative matrix factorization (NMF). workflow applied to the scRNA-seq count matrix to extract gene expression programs (GEPs). (H) Distribution of module scores corresponding to the recurrent program RP2 across cells from each PDO. Primary PDOs are shown in gray, and metastasis PDOs are shown in red.

Supplementary Figure 2

A

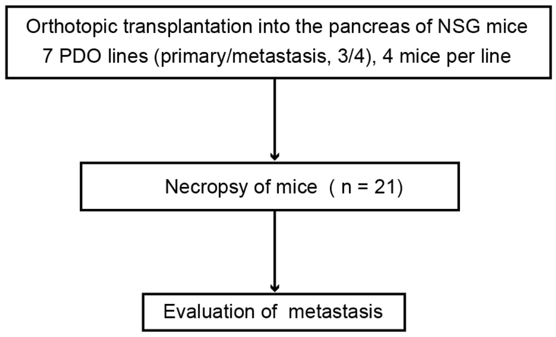

B

	No.1	No.2	No.3	No.4
PDOX02	M	E	E	E
PDOX03	E	E	M	E
PDOX09	E	D	E	E
PDOX10	E	N	E	N
PDOX11	D	E	M	M
PDOX12	M	E	D	D
PDOX16	D	M	D	D

C

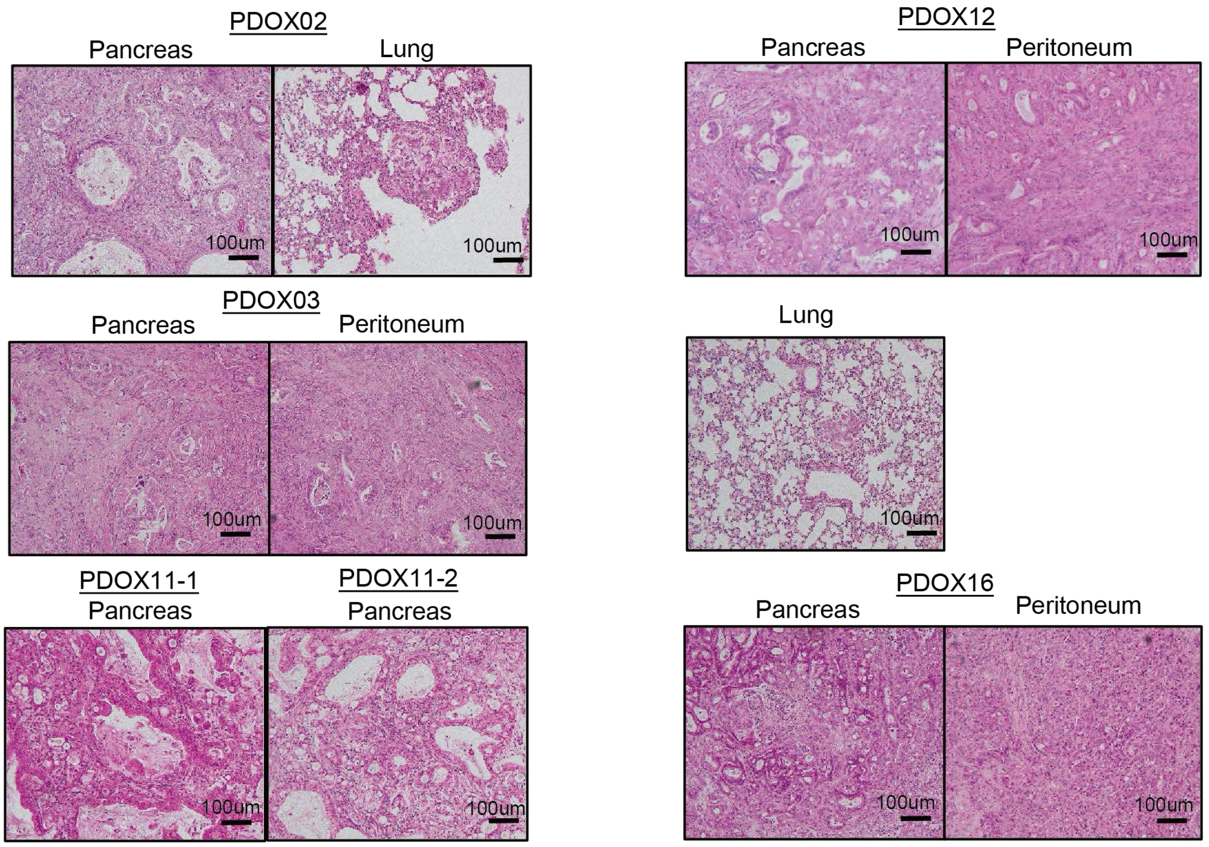

Supplementary Figure 2. Results of orthotopic transplantation of seven PDO lines and histopathological analysis of tumors in NSG mice, related to Fig. 3. (A) Flowchart of orthotopic transplantation of seven PDO lines into the pancreas of NSG mice. (B) Necropsy results of 22 mice. "D" indicates mice that died before necropsy. "E" indicates that engraftment was observed and that distant metastasis was not observed. "M" indicates that distant metastasis was observed. "N" indicates that engraftment was not observed. (C) Histopathological images of primary pancreatic and metastatic lesions in seven patient-derived orthotopic xenografts (PDOXs) (PDOX02, PDOX03, PDOX11, PDOX12, and PDOX16).

Supplementary Figure 3

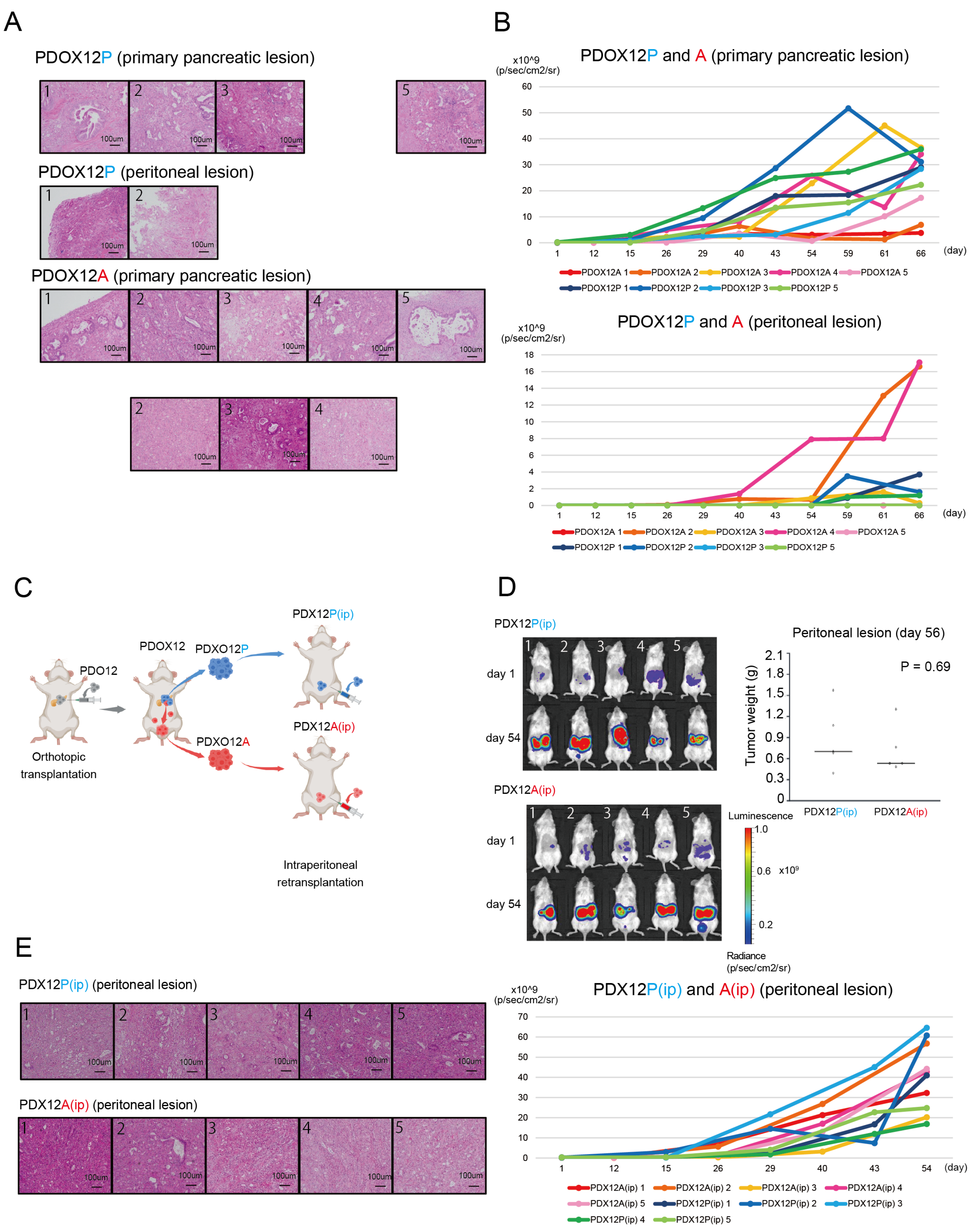

Supplementary Figure 3. Histopathological images of PDOX12P and PDOX12A, and differences in the proliferative capacity between PDXO12P and PDXO12A in the peritoneum *in vivo*, related to Fig. 3. (A) Histopathological images of primary pancreatic and peritoneal metastatic lesions in PDOX12P and PDOX12A, respectively. The numbers in the *in vivo* imaging system (IVIS) images correspond to those in the histopathological images.

PDOX12P (No. 4) died prior to necropsy. Hence, histological analysis could not be performed. (B) Longitudinal changes in IVIS bioluminescence signals of primary pancreatic (top) and peritoneal metastatic (bottom) lesions in PDOX12P and PDOX12A. (C) Intraperitoneal retransplantation scheme of organoids derived from the primary pancreatic lesion (PDOX12P) or peritoneal metastases (PDXO12A). (D) *In vivo* imaging and tumor burden analyses of PDXO12P- and PDXO12A-derived intraperitoneal patient-derived xenografts (PDXs) (PDX12P(ip) and PDX12A(ip), respectively). Representative IVIS bioluminescence image of PDX12P(ip) (top left) Representative IVIS image of PDX12A(ip) (middle left). Dot plot comparing the weights of peritoneal lesions between PDX12P(ip) and PDX12A(ip) (top right). Each dot represents an individual mouse, and horizontal bars indicate median values. Statistical significance was assessed using the Mann–Whitney U test. Longitudinal changes in IVIS bioluminescence signals of peritoneal lesions in PDOX12P(ip) and PDOX12A(ip) (bottom). (E) Histopathological images of peritoneal lesions in PDX12P(ip) and PDOX12A(ip). The numbers in the IVIS images correspond to those in the histopathological images.

Supplementary Figure 4

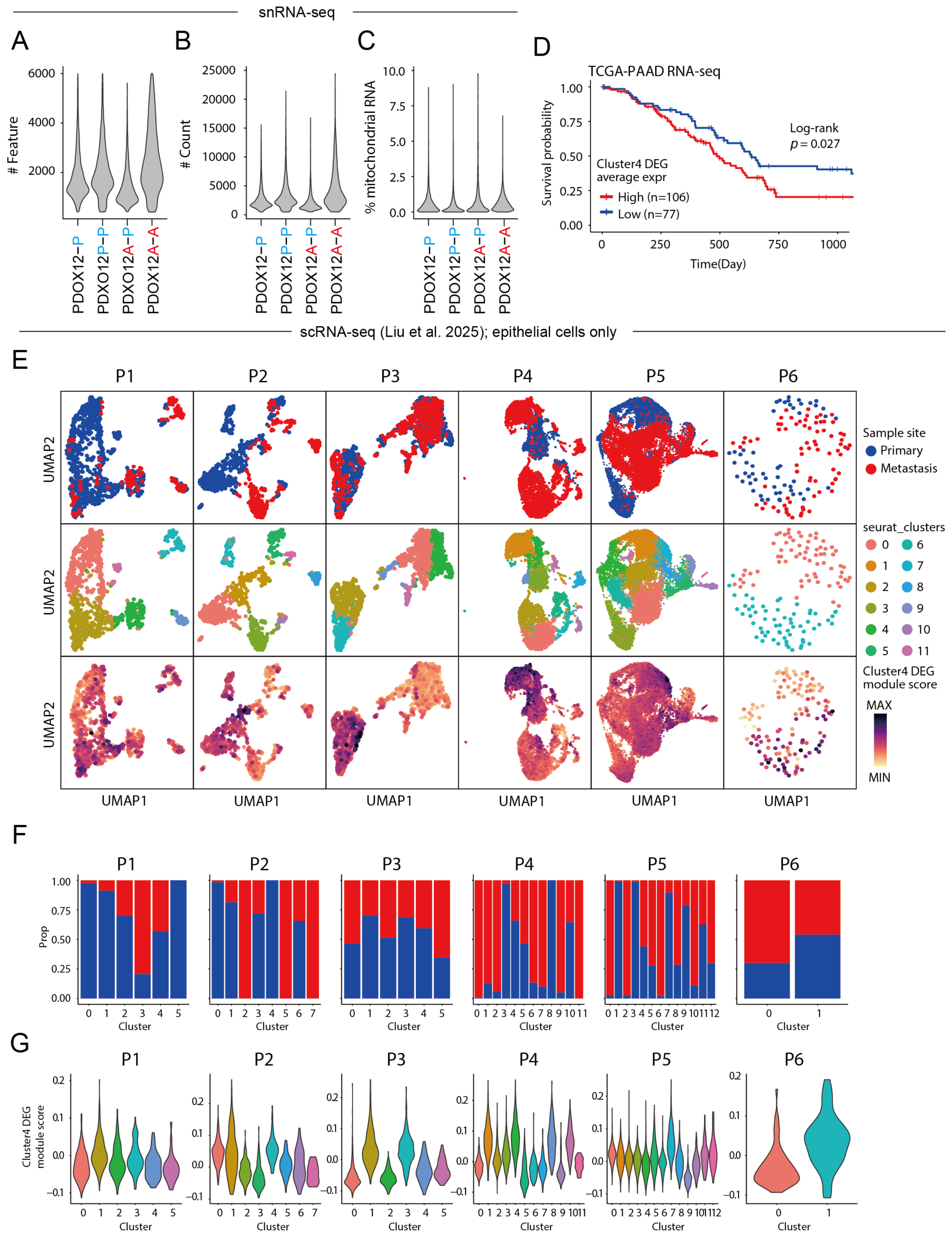

Supplementary Figure 4. Single-nucleus RNA (snRNA-seq) sequencing data of PDOX samples, related to Fig. 4 and *clinical relevance of cluster 4 gene program*. (A–C) Quality control metrics for all snRNA-seq samples. Distribution of detected features per nucleus (A), distribution of total UMI counts per nucleus (B), and percentage of mitochondrial RNA counts

per nucleus (C). Each violin plot represents the distribution of individual nuclei within each sample. (D) Kaplan–Meier overall survival curves of patients with pancreatic ductal adenocarcinoma (TCGA-PAAD cohort), stratified into high and low groups based on the average expression score of cluster 4–specific genes. (E) Reanalysis of publicly available single-cell RNA-seq data from paired primary pancreatic tumors and matched liver metastases (six patient pairs; Liu et al., 2025), focusing on epithelial cells only. UMAP embeddings are shown, colored by sample origin (top), Seurat-defined clusters (middle), and module scores of the cluster 4 DEG gene set (bottom). (F) Proportional representation of epithelial cells derived from primary tumors and liver metastases within each Seurat-defined cluster across the six patient pairs. (G) Violin plots showing the distribution of cluster 4 DEG module scores across individual clusters in each patient pair.

Supplementary Figure 5

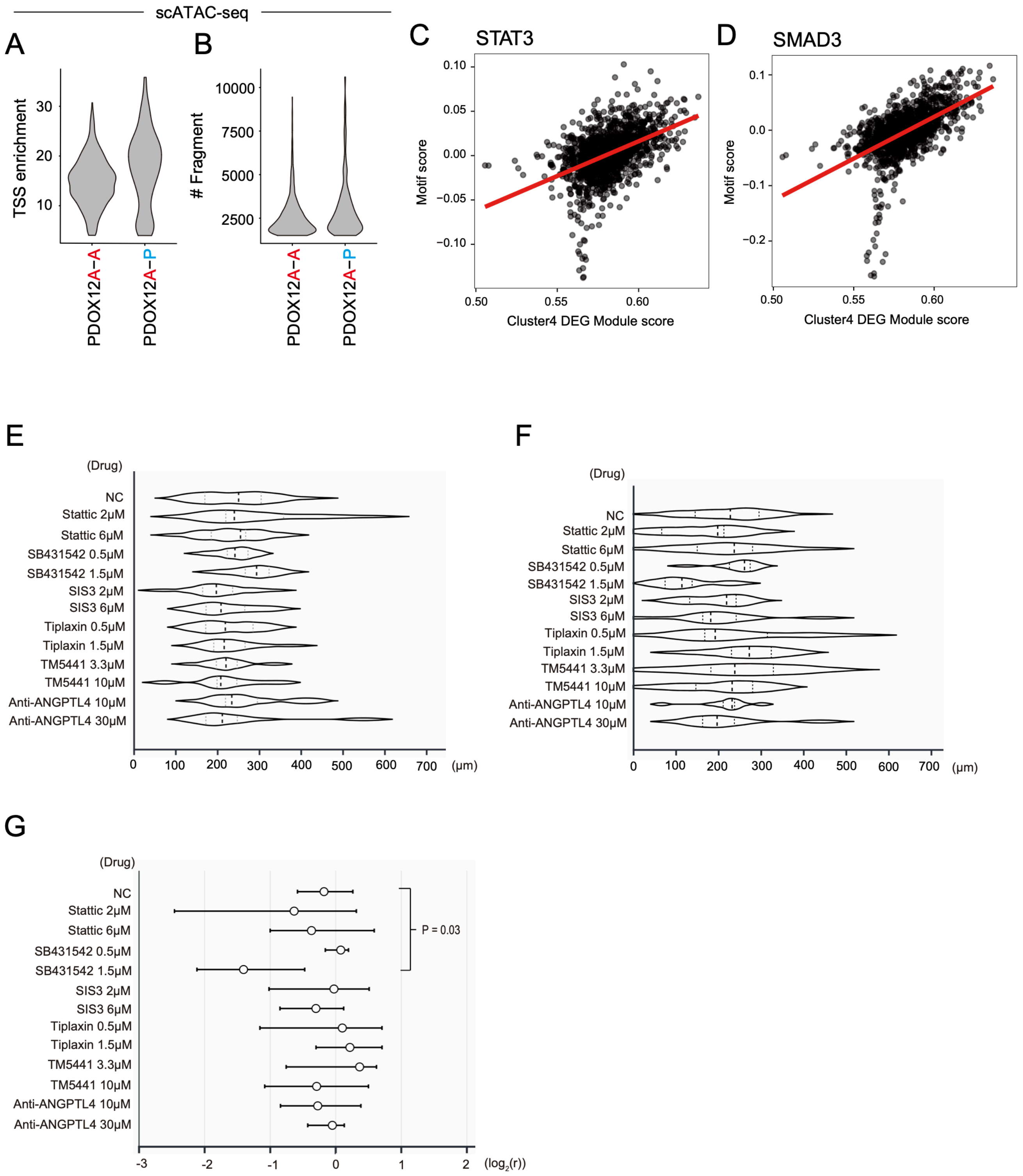

Supplementary Figure 5. Single-cell ATAC sequencing (scATAC-seq) data of PDOX samples, related to Fig. 4 and *in vitro* migration assay. (A, B) Quality control metrics for scATAC-seq data. Distribution of transcription start site (TSS) enrichment scores per cell (A) and distribution of the number of unique fragments per cell (B). (C, D) Example scatter plots showing correlations between the cluster-4-specific DEG module score and motif activity scores for STAT3 (C) and SMAD3 (D) at the single-cell level. (E) Violin plot of PDXO12A migration distance during 0–24 hours under each drug condition (black dashed line, median;

gray dashed lines, 25th and 75th percentiles). (F) Migration distance during 72–96 hours. (G) Bar plot showing the $\log_2$ migration distance ratio (r) of PDXO12A under each drug condition. Bars indicate the 95% confidence interval, and white dots represent the median.

Supplementary Table 1. Details of the six mice that developed metastases.

Supplementary Table 1. Details of the six mice that developed metastases.

Organoids	Mice	Sample	Metastatic sites	Detection by fluorescence imaging	Organoid re-establishment from metastatic sites
PDO02	PDOX02	pancreatic lesion	pul	No	No
PDO03	PDOX03	pancreatic lesion	peri	Yes	No
PDO11	PDOX11-1	ascites	peri	No	Yes
PDO11	PDOX11-2	ascites	peri	Yes	Yes
PDO12	PDOX12	ascites	peri/pul	Yes/No	Yes/No
PDO16	PDOX16	pleural effusion	peri	Yes	Not performed

peri; peritoneum, pul; lung

Supplementary Table 2. Details of peritoneal metastasis in PDOX12P and PDOX12A.

Supplementary Table 2. Details of peritoneal metastasis in PDOX12P and PDOX12A.

PDOX	No.	The number of lesions	The luminescence intensity of lesions ($x10^9$ p/sec/cm2/sr)	PCI	Tumor weight (g)
PDOX12P	1	1	371.7	3	0.03
PDOX12P	2	1	161.8	3	0.04
PDOX12P	3	0	0	0	0
PDOX12P	4	NA	NA	NA	NA
PDOX12P	5	0	0	0	0
PDOX12A	1	0	0	0	0
PDOX12A	2	3	1659.8	8	0.28
PDOX12A	3	1	27.8	3	0.03
PDOX12A	4	2	1710.8	6	0.13
PDOX12A	5	0	0	0	0

PCI; peritoneal carcinomatosis index, NA: not available

Supplementary Data 1. Top 200 gene lists of 108 GEPs identified by cNMF across 17 PDOs (related to Figure 2C).

Supplementary Data 2. Gene lists of 8 RPs derived from cNMF analysis (related to Figure 2C).

Supplementary Data 3. DEG lists for each cluster identified in single-nucleus RNA sequencing (related to Figure 4E).

Supplementary Data 4. Correlation between DEG module scores and motif activity scores (related to Figure 4J).

Supplementary Data 5. Raw migration distances and derived migration ratios (r and log2[r]) for organoid migration assays (related to Supplementary Figure 5E–G).
